# Chronic Diarrhea

**Published:** 1850-11

**Authors:** 


### CHRONIC DIARRHEA.

This affection is often troublesome, and frequently resists all ordinary means of relief. We have recently seen, in the "Extracts from the Records of the Merrimack County Pathological Association," published in the *New Hampshire Journal of Medicine*, the *lapis divinus* strongly recommended in the treatment of Chronic Diarrhea. This article was introduced to the notice of English physicians, by Mr. Curtis, as an excellent remedy for otorrhea, and is unquestionably useful in that annoying malady. In the Records, referred to above, Dr. Moore reports the successful treatment of chronic diarrhea, with the "lapis divinus." It is made of sulphate of copper, nitrate of potass, and alum, of each sixteen parts—powder them separately, and mix them well together; fuse them in a glass vessel in a sand bath, and add one part of powdered camphor; break the mass in pieces when cold.

For otorrhea, two drachms of the "lapis divinus" are dissolved in an ounce of distilled water; a drachm of this solution is added to six ounces of rose or distilled water, which constitutes the proper strength for an injection in otorrhea. It is injected into the meatus twice a day. The strength of the injection is gradually increased, as the patient can bear it.

For diarrhea, Dr. Moore recommends fifteen drops, of the first solution mentioned above, three times a day. It does not nauseate.

Many cases of what are called Mexican diarrhea, yet linger in the land, and many cases of undoubted indigenous origin, and we hope the "lapis divinus" may have a fair trial given of its powers. We shall be pleased to hear from any of our correspondents who may try the remedy, so that a proper estimate may be placed upon it. B.

